# Supplementary material for: Electrochemical Measurement of Interfacial Distribution and Diffusion Coefficients of Electroactive Species for Ion-Exchange Membranes: Application to Br2/Br− Redox Couple
Source: Membranes (Basel). 2022 Oct 26;12(11):1041. doi: 10.3390/membranes12111041 (PMC9693329; doi:10.3390/membranes12111041)
Supplement: Supplementary file 1 [file membranes-12-01041-s001.zip › membranes-1975443-supplementary.pdf]

Supplementary materials

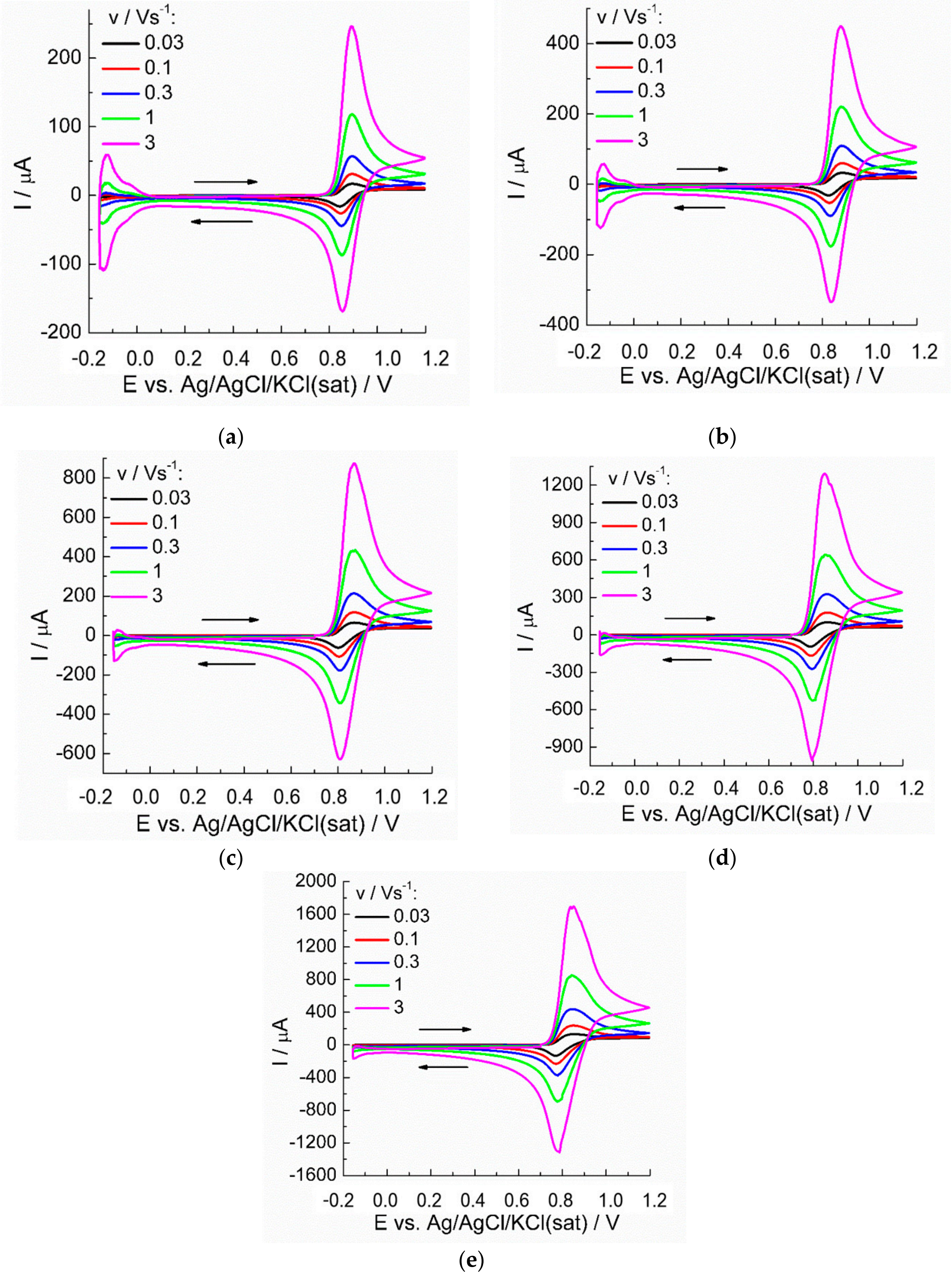

Figure S1. Cyclic voltammograms for Pt electrode (diameter: 1 mm) coated with Nafion 212 membrane in contact with 2 M sulfuric acid with addition of HBr, c^0: 0.125 M (a), 0.25 M (b), 0.5 M (c), 0.75 M (d), 1 M (e). Scan rates indicated in the figures. First cycles.

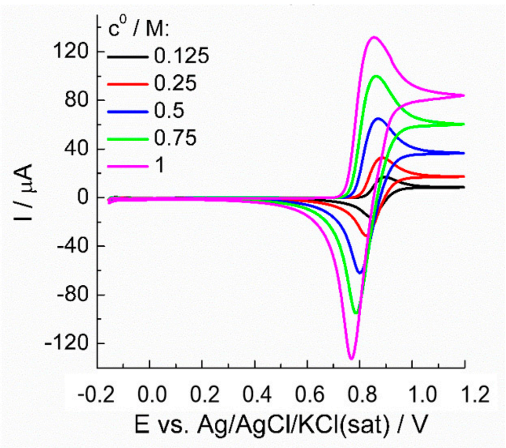

(**a**)

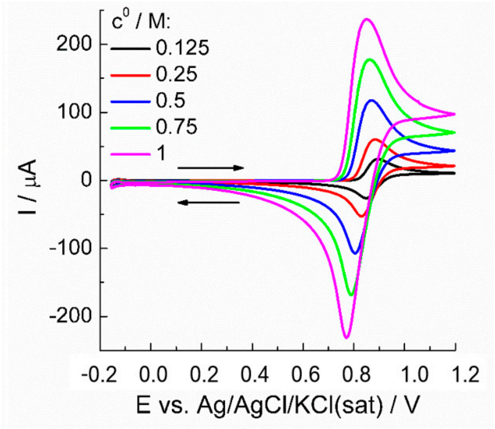

(**b**)

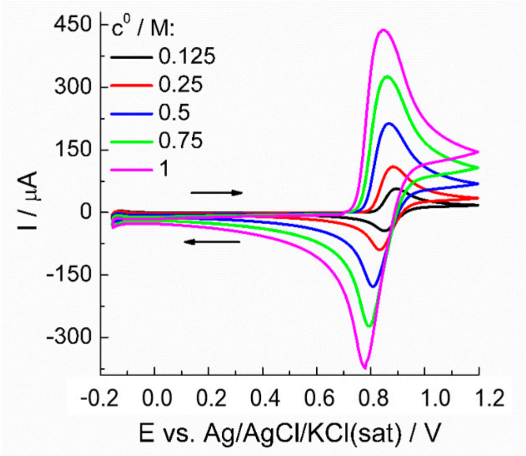

(**c**)

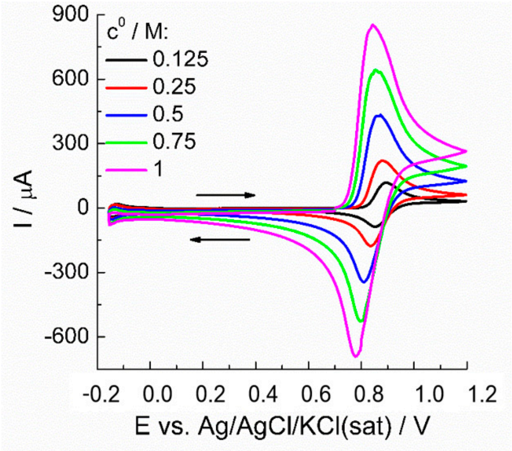

(**d**)

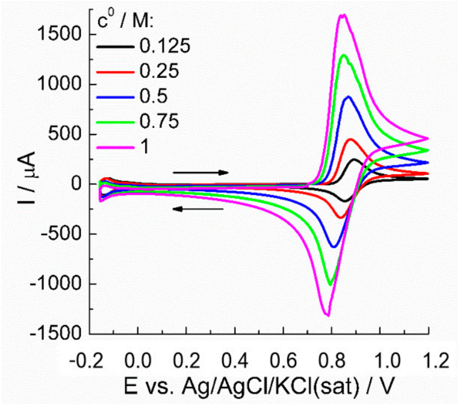

(**e**)

Figure S2. Cyclic voltammograms for Pt electrode (diameter: 1 mm) coated with Nafion 212 membrane in contact with 2 M sulfuric acid with addition of HBr for a set of scan rates: 0.03 V/s (a), 0.1 V/s (b), 0.3 V/s (c), 1 V/s (d), 3 V/s (e). Concentrations of HBr, c^0, indicated in the figures. First cycles.
